# New Middle to ?Late Jurassic dinosaur tracksites in the Central High Atlas Mountains, Morocco

**DOI:** 10.1098/rsos.231091

**Published:** 2023-09-27

**Authors:** Ahmed Oussou, Peter L. Falkingham, Richard J. Butler, Khadija Boumir, Driss Ouarhache, Kawtar Ech-charay, André Charrière, Susannah C. R. Maidment

**Affiliations:** ^1^ GERA Laboratory, Faculty of Sciences Dhar El Mahraz, Sidi Mohamed Ben Abdellah University, Fez, Morocco; ^2^ School of Biological and Environmental Sciences, Liverpool John Moores University, Liverpool, UK; ^3^ School of Geography, Earth and Environmental Sciences, University of Birmingham, Birmingham, UK; ^4^ Toulouse III University, Anduze, France; ^5^ Fossil Reptiles, Amphibians and Birds Section, Natural History Museum, London, UK

**Keywords:** photogrammetry, trackways, dinosaur, Isli Formation, High Atlas, Morocco

## Abstract

Besides bones, fossil tracks and trackways are important sources of knowledge about dinosaur palaeobiology. Here, we report three new tracksites from two different synclines in the Imilchil area, Central High Atlas, Morocco. The tracks and trackways are preserved in fluvial deposits in different levels of the Isli Formation (Early Bathonian–?Upper Jurassic), and contain impressions made by sauropods, theropods and ornithopods, as well as tracks that might represent bird-like non-avian theropod dinosaurs. In addition to traditional field measurements, three-dimensional digital models of the track sites were created using photogrammetry. These new tracksites add to the rich faunal ichnoassemblage already recorded from the High Atlas Mountains and North Africa, which is considerably richer than the contemporaneous body fossil record, and also provide new data on dinosaurs–substrate interactions.

## 1. Introduction

The Imilchil area is located in the heart of the Central High Atlas, and is the richest region for dinosaur and other vertebrate traces in Morocco. These traces are preserved on the surfaces of beds in the Imilchil (Upper Bajocian–Lower Bathonian) and Isli (Lower Bathonian–?Upper Jurassic) formations in the various synclines of the region. The tracksites described here are preserved in the Isli Formation. This formation consists of a thick succession of fluviolacustrine red bed deposits. The documentation of the tracks of the Imilchil region is relatively recent, beginning with Gierliński *et al.* [1] who studied three tracksites on the northern limb of the Ait Ali Ou Ikkou (AAK) syncline. Those authors described theropod footprints of small (*Wildeichnus* sp.), medium (*Jialingpus* sp. and *Carmelopodus*) and larger size (*Changpeipus* sp., *Therangospodus* sp. and *Dinehichnus* sp.), as well as those of sauropods (*Parabrontopodus* sp.) and an ornithiscian track (*Stegopodus* sp.). Subsequently, Gierliński *et al.* [2] described bird-like traces (*Trisauropodiscus* isp.) in the upper part of the Imilchil Formation on the northern limbs of the AAK syncline and the Plateau des Lacs syncline. Klein *et al.* [3] and Masrour *et al.* [4] studied crocodilian (*Crocodylopodus meijidei* and *Batrachopus* isp.) footprints in the Isli Formation of the northern limb of the AAK syncline. Klein *et al.* [3] also reported the coexistence of pterosaur (*Pteraichnus*) and crocodilian (*Crocodylopodus meijidei*) footprints. Oukassou *et al*. [5] studied tracksites including large sauropod tracks referred to as the ichnospecies *Polyonyx* isp. Masrour *et al.* [6] documented the tail tracks of swimming crocodiles. Ceniceros *et al.* [7] described two very large isolated colossal theropod footprints with lengths exceeding 70 cm. Finally, Klein *et al*. [8] reviewed previously described footprints and documented new tracksites in beds of the Imilchil and Isli formations; those authors described new footprints that they attributed to multiple theropod ichnogenera (*Carmelopodus*, *Wildeichnus*, *Changpeipus*, *Kayentapus*, *Megalosauripus*, *Trisauropodiscus* and Grallatoridae), an ornithopod ichnogenus (small *Dinehichnus*?) and large ornithischian tracks (*Sinoichnites*).

Studies of wider dinosaur ichnology in Morocco began with the work of Plateau [9], and research has increased during the last two decades with several papers devoted to Jurassic tracksites, especially in areas geographically close to Imilchil (e.g. [10–20]).

Taken together, these works demonstrate a diverse vertebrate assemblage in the Middle to Late Jurassic of Morocco which is not replicated in the body fossil record of the area. The main objective of this work is to document three new tracksites in two synclines (AAK and Outerbat) of the Imilchil region. These tracksites shed further light on Jurassic dinosaur communities and the palaeoenvironmental reconstruction of this period in the study area.

## 2. Geological setting

**Figure 1.**
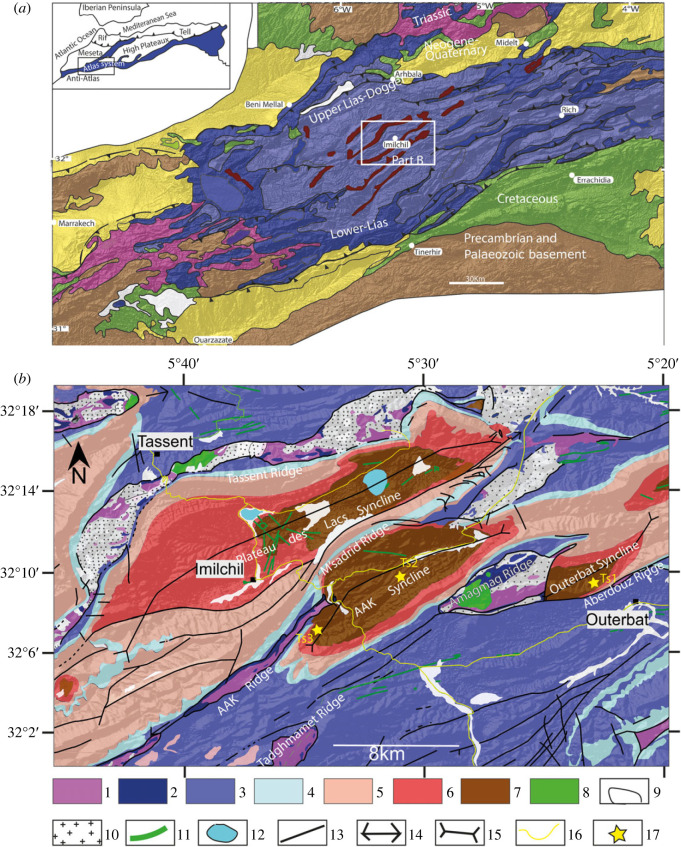
Geological setting of the study area. (*a*) Imilchil area in its regional geological context (from the geological map of Morocco at 1 000 000 and Teixell *et al*. [21]). (*b*) Detailed geological map of the Imilchil area (from the geological maps at 100 000 of Imilchil, Tinghir, Tinjdad and Tounfit, modified). For the stratigraphic terms of (*b*): 1, CAMP basalts and argillites Triassic; 2, Lower Lias limestones; 3, Tassent Formation; 4, Bab n'Ouayad Formation; 5, Tislit Formation; 6, Imilchil Formation; 7, Isli Formation; 8, Tasraft Formation; 9, Quaternary deposits; 10, Jurassic-Cretaceous magmatic intrusions; 11, doleritic dykes; 12, lakes; 13, faults; 14, anticlinal axis; 15, synclinal axis; 16, roads; 17, studied tracksites.

The Imilchil-Outerbat region is located in the heart of the Central High Atlas and belongs to the Atlas system, which is an intracontinental alpine belt (figure 1*a*; [22,23]). The chain extends from the central Atlantic to Tunisia and was formed as a result of the inversion of Triassic–Early Jurassic rifting, in response to the convergence of the African, Iberian and Eurasian plates since the end of the Cretaceous (e.g. [23–25]).

The Imilchil-Outerbat region is a mountainous area characterized by a succession of narrow anticlinal ridges and synclinal gutters which are generally trending 60° N (figure 1*b*). The core of the ridges is occupied by Central Atlantic Magmatic Province (CAMP) green basalts and latest Triassic red pelites [26–29], as well as a transitional series of Upper Jurassic–Lower Cretaceous magmatic intrusions (e.g. [30–34]. Some of the ridges are unconformably overlain by red pelites and basaltic flows of the Palaeogene Tasraft Formation [35,36]. The synclines are filled by deposits of Middle Jurassic age, organized in a mega-sequence of marine formations, surmounted by a thick continental series.

Stratigraphically, the terraines of the Central High Atlas are more than 6000 m thick and were originally subdivided into a marine formation (the Agoudim Formation) overlain by a continental formation (the Anmzi Formation) [37].

**Figure 2.**
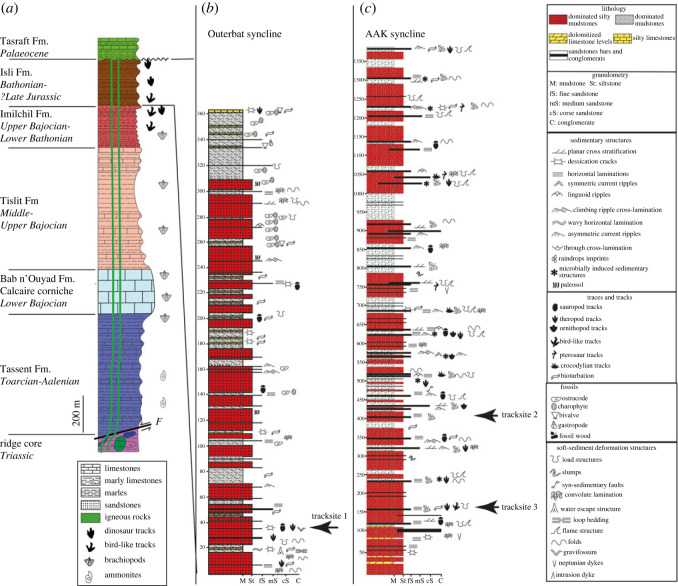
The situation of tracksites levels in the Jurassic series of the Imilchil region. (*a*) Synthetic log of the geological formations of the Imilchil region [38,39]. (*b*) Sedimentological log of the Outerbat syncline. (*c*) Sedimentological log of the AAK syncline.

However, Charrière *et al.* [38] established a new and more detailed subdivision for the terraines of the Imilchil area (figure 2*a*). From the base to the top, these are: (i) the Tassent Formation (Toarcian–Aalenian) formed by 600 m of carbonates, siltstones and marls; (ii) the Bab n'Ouyad Formation (Lower Bajocian) formed by 200 m of reef limestones; (iii) the Tislit Formation (Upper Bajocian) formed by 500 m of marls and limestones and shallow water limestones; (iv) the Imilchil Formation (Upper Bajocian–Lower Bathonian), formed by 200 m of marine sandstone marls and calcarenites with intercalations of continental siltstones and sandstones; and (v) the Isli Formation (Lower Bathonian–?Upper Jurassic) consisting of 1500 m of fluvio-lacustrine deposits. These continental deposits are quite rich in dinosaur and other vertebrate footprints ([8] and references therein).

The three dinosaur tracksites studied in this work are located in the Isli Formation of the Outerbat and Ait Ali Ou Ikkou synclines. The Outerbat syncline is bordered by Amagmag ridge on the west side and Aberdouz ridge on the east. Ait Ali Ou Ikkou syncline is bordered by Msadrid and Ait Ali Ou Ikkou ridge on the northwestern side and the Amagmag ridge to the southeast (figure 1*b*).

The age of the Isli Formation remains poorly defined. The formation has been attributed to the Bathonian–Callovian [38,40] on the basis of brachiopods from the upper marine beds of Imilchil Formation (underlying the Isli Formation) in the Plateau des Lacs syncline. The age of the Isli Formation has been suggested to be as young as Late Jurassic based on ichnotaxa [8].

Recently, we collected brachiopods in the uppermost beds of the marine limestones of the Imilchil Formation in the Outerbat and AAK synclines. These brachiopods are represented by two species: *Burmirhynchia athiensis* (Rousselle, 1965) and *Cymatothynchia reynesi* (Oppel, 1865), which suggests a basal Bathonian age, zigzag zone, Parvum sub-zone (Y. Alméras (deceased) from the University Claude-Bernard-Lyon 1 and P. Fauré from the Museum of Natural History of Toulouse 2022, personal communication). This would support a Bathonian to Late Jurassic age for the overlying Isli Formation, in which the tracks described here are preserved.

## 3. Methods

### 3.1. Track documentation

The sites described herein were documented via photogrammetry according to accepted standard ichnological protocols [41]. Photographs were taken in the field using a Nikon D750, and processed into textured meshes using Reality Capture v. 1.2. Digital models were produced of track surfaces, and of individual tracks separately. Height maps were generated using Blender. All data (photographs and models) are available from https://doi.org/10.6084/m9.figshare.23374418.

### 3.2. Track measurement

In order to study the different tracks in detail, make interpretations about the trackmaker and to make comparison with previous discoveries, field measurements of footprints remain a fundamental tool. Measurements taken of sauropod, ornithopod and theropod footprints in the field were the length (L) of each track, its width (W), the distances between successive tracks (PL) and the angles between the digits of tridactyl prints II^III, III^IV and II^IV.

### 3.3. Palaeoenvironmental interpretation

**Figure 3.**
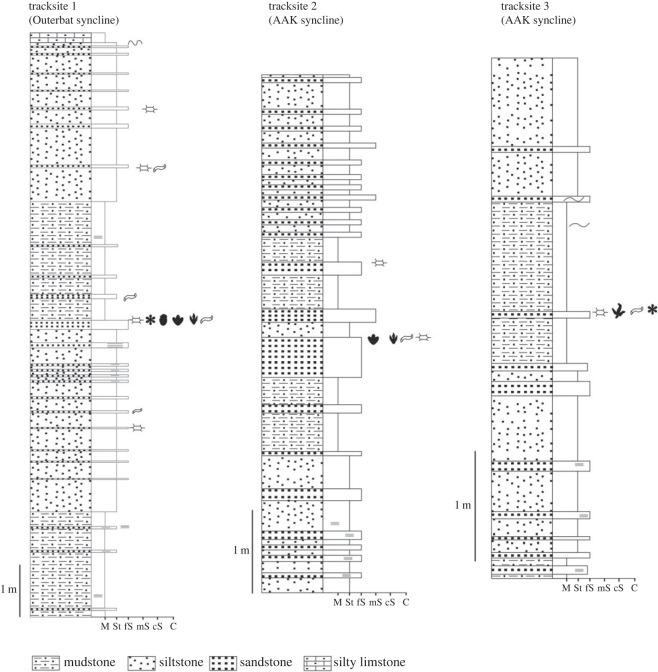
The detailed sedimentological logs of the different studied tracksites. N.B. the symbols of the sedimentary structures and tracks are the same as those used in figure 2.

Stratigraphic sections of the entire Isli Formation within the two synclines (figure 2), and detailed sections at the tracksites (figure 3), were studied. Unconsolidated sediments were washed for microfossils, and thin-sections were made of indurated rocks and examined under a petrographic microscope.

## 4. Results

### 4.1. Tracksite one

#### 4.1.1. Sedimentology and palaeoenvironmental interpretation

Tracksite one is located on the southern limb of the Outerbat syncline. It is the first discovery of tracks in the Outerbat syncline. The tracks, particularly larger impressions, are generally moderately well preserved (see [42] and [43]) on the surface of a reddish fine sandstone bed (figure 3). Most tracks score 1–2 on Belvedere and Farlow's [44] quality scale. This centimetric bed is intercalated with a series of clayey and silty deposits. It corresponds to the top of an alternation of almost 5 m of silty deposits and fine red sandstone forming a horizontal heterolithic level. It is also overlain by siliciclastic facies comprising grey mudstones and siltstones intercalated with sandstone layers over a thickness of 6 m, and the sequence is capped by silty limestones.

These siltstones and mudstones correspond to alluvial and lacustrine floodplain deposits that extend across the syncline. The sandstone beds are unchannelled and unconfined fluvial sheetflood deposits. The whole package corresponds to the fluvio-lacustrine sequence of the Isli Formation in the Outerbat syncline. This sequence is capped by lacustrine limestones with charophytes and lacustrine ostracods.

#### 4.1.2. Description of the track horizon

**Figure 4.**
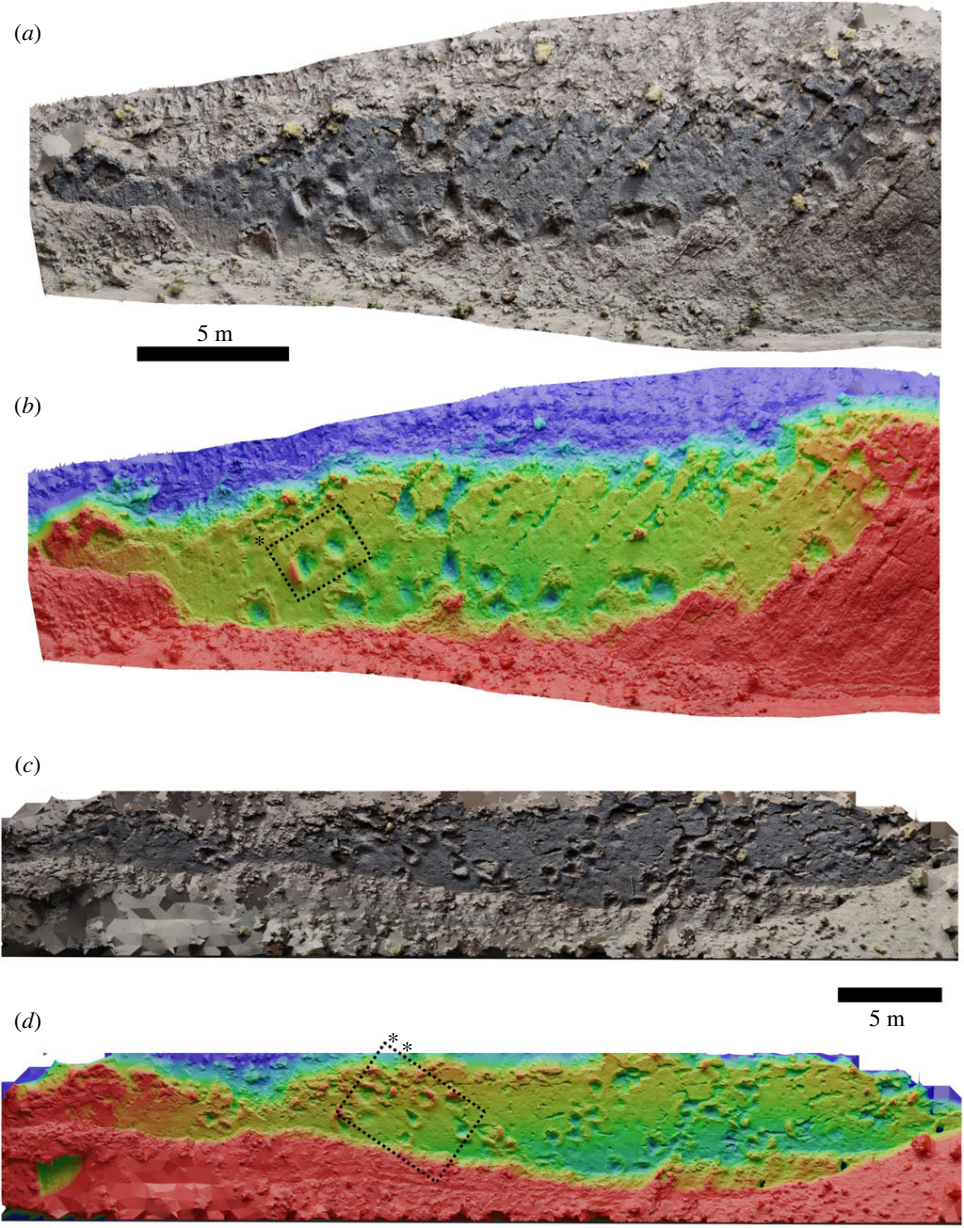
Both surfaces of tracksite 1, presented as photogrammetric models in true-colour and height-map. The two surfaces are located some distance apart, separated by a valley, but belong to the same bedding surface. Red–blue scale in both *b* and *d* is 50 cm. Boxes * and ** are shown in detail in figure 5*a–c*.

This track horizon is the first to be described in the Outerbat syncline and is located 1.5 km northwest of Outerbat village (coordinates: 32°8′59.06″ N; 5°22′40.73″ W). The trampled surface is a single bedding plane that is divided into two halves by a small valley (figure 4). The beds are inclined at an angle of 40° to the northwest. The two halves of the track horizon are more than 60 m long and each up to 4.5 m wide.

The track horizon is a trampled surface comprising numerous trackways that cross each other. Eleven distinct theropod trackways (with 34 tracks in total), six sauropod trackways, one ornithopod trackway and three trackways that may correspond to thyreophoran dinosaurs are present on the bedding plane, along with a number of isolated tracks. The ornithopod track trends 226° and comprises 19 tridactyl tracks with digit impressions that are sub-equal in length. Each footprint is approximately 30 cm long from heel to the end of the central digit, and the heel–heel stride length is approximately 85 cm. The stride length does not appear to vary significantly along the trackway. We studied an area of the bedding plane where three sets of theropod trackways cross-cut each other. All of the trackways consisted of tridactyl footprints with narrow digit traces and pointed ends, and the central digit was the longest. The largest trackway trended 266°. It comprised three tracks with a footprint length of approximately 35 cm, and a heel–heel stride length of approximately 112 cm. A second trackway, comprising smaller footprints, cross-cut the larger one and trended 121°. It is composed of four tracks, each about 30 cm in length and with a stride length of approximately 100 cm. The smallest track comprised three tracks trending at 166°. The tracks were approximately 26 cm long and the heel–heel stride length was 106 cm. The longest theropod trackway observed comprises 10 distinct tracks, and trends 094°. Each footprint is approximately 26 cm in length, and the heel–heel stride length is approximately 85 cm. The largest theropod trackway preserved comprises five tracks with a footprint length of approximately 39 cm and a heel–heel stride length of approximately 100 cm.

Sauropod tracks on the surfaces range from approximately 50 to 80 cm in length, with similar width measurements, and appear as rounded or elliptical impressions surrounded by displacement rims (maximum zone of deformation; [45]). Size including displacement rims is of the order of 1.3–1.4 m. Tracks appear as larger and smaller impressions, which may indicate manus and pes; however, they do not appear to be clearly arranged in consistent trackway patterns, primarily because of the narrow exposure of each surface, though linear relations between groups of sauropod tracks are observed (figure 4*c,d*). Similar sauropod tracks have already been documented in the AAK syncline [5,7,8,46], but this is the richest track horizon in terms of the number of sauropod footprints in the entire region.

**Figure 5.**
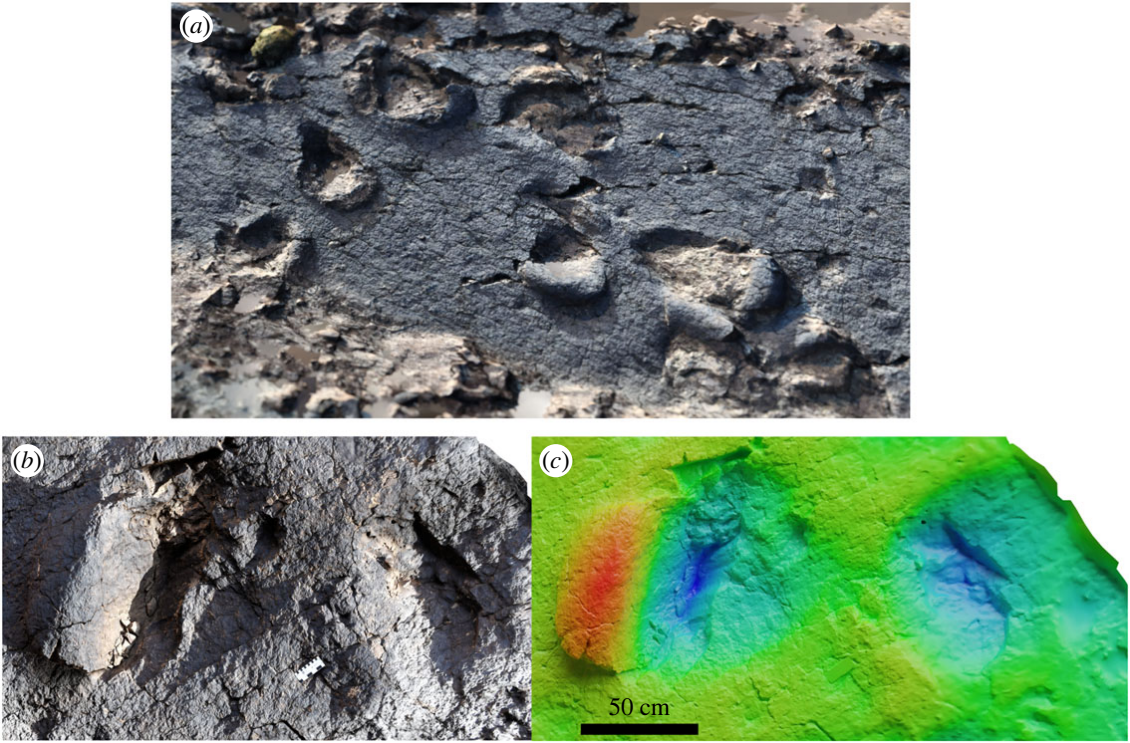
(*a*) Close-up of box ** in figure 4, in isometric view, displaying several sauropod tracks with displacement rims occurring in the same direction for each. (*b*), (*c*): close ups of track * in figure 4 in true colour and height map, respectively (red–blue = 30 cm), showing how the raised area of sediment in front of the track remains coherent, slipping at the sides.

Of particular note are the raised displacement rims around the sauropod tracks. Several tracks indicate large-scale movement of substrate under the moving foot, in such a way that the original surface sheared as it was pushed upwards, remaining relatively intact (figure 5). The sediment surface at the time of track formation was therefore relatively competent and probably overlaid a softer material below, as would be found when the surface of a muddy area has dried in the sun, but the subsurface remains soft. Many of these displacement rims have been weathered or eroded and now have a blocky appearance. The smaller theropod tracks on the surface do not have observable displacement rims, and the interiors are blocky and do not retain anatomical features such as skin or pad impressions, further consistent with a drier, more competent sediment surface.

### 4.2. Tracksite two

#### 4.2.1. Sedimentology and palaeoenvironmental interpretation

The tracksite is located on the southern limb of the AAK syncline, in the middle part of the Isli Formation. The latter is formed in this syncline by more than 1500 m of fluvio-lacustrine deposits (figure 2*c*). Like the tens of other dinosaur footprint sites in the AAK syncline, the footprints of this tracksite are well preserved on the surface of a massive greyish fine sandstone bed. It is a centimetric sandstone bed of decametric extension, intercalated between grey and reddish mudstones and siltstones with an intercalation of fine sandstone with horizontal heterolithic stratifications. The grey silty mudstones have yielded charophyte oogonia and limnic ostracods. The sandstone bars are also rich in fossilized wood, ripples and invertebrate bioturbation. The palaeoflora and palaeofauna of these deposits indicate a continental depositional environment that corresponds to the alternation of large ephemeral lakes and distal sandstone channels of kilometre-wide extension.

#### 4.2.2. Description of the track horizon

**Figure 6.**
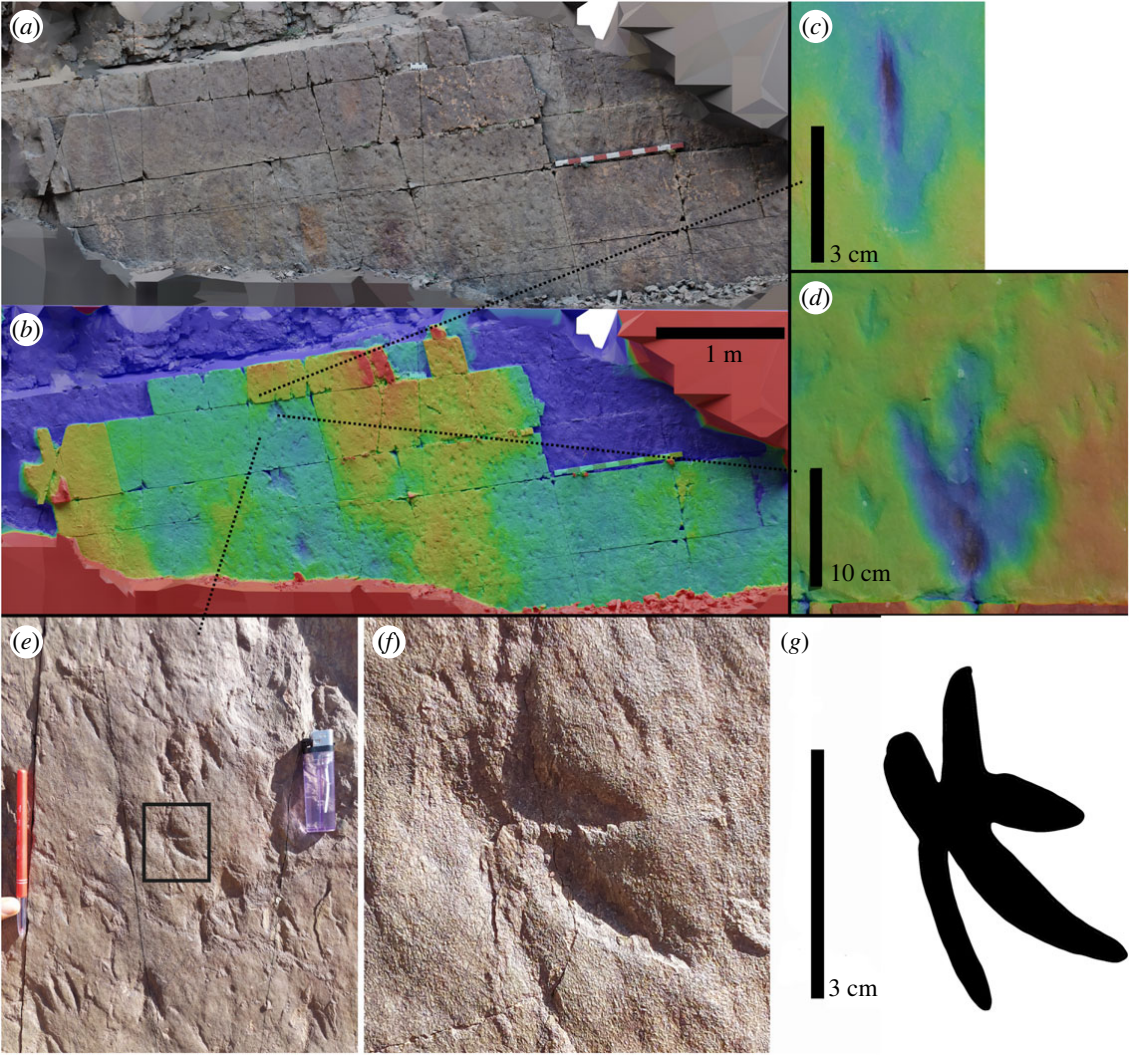
Tracksite two, displaying numerous small tridactyl tracks, and one larger tridactyl track, all heading in the same direction (top–bottom in this image). Presented as true-colour (*a*) and height-map (*b*). Red-blue scale = 5 cm. Close up views of a theropod track (*c*) and ornithopod track (*d*) are presented as height-mapped renders (colour scale arbitrarily changed from *b* for clarity). Panels (*e*–*g*) show wide (*e*), close (*f*) and interpretive (*g*) images of the putative pterosaur track.

This site presents a trampled surface, and was uncovered by the work of villagers collecting stones for house construction, which unfortunately destroyed several other surfaces in this syncline. The track horizon is located 1 km south of Taghighacht village (coordinates: 32°9′35.76″ N; 5°30′35.71″ W). The sandstone slab bearing the footprints is almost 10 m long and up to 2.5 m wide, and dips about 70° to the north. The surface is marked by numerous small, tridactyl theropod trackways, one putative pterosaur track, and one ornithopod trackway (figure 6). The tridactyl tracks have pointed digit traces with an elongate central toe, and are 3.8–5.5 cm long and 2–2.5 cm wide. The angles between the digits of these prints are approximately: II^III = 19.5°, III^IV = 28.68° and II^IV = 48.18°. All of the trackways trend in the same direction, at approximately 270°. Stride lengths vary along each trackway, and from time to time, the tracks appear next to each other, as though the animals were stopping, standing and then starting to move again.

The tracks on this surface are the smallest tridactyl footprints found in the region, and even in the whole country. In this area, Klein *et al*. [8] documented two small isolated mesaxonic pes tracks assigned to *Wildeichnus* ichnogenus. These tracks are larger (3.5–5.3 cm) than those described here. Klein *et al*. [3,8] have also reported the presence of pterosaur footprints in the Isli Formation.

The putative pterosaur track is presented in figure 6. The impression appears to display four digits asymmetrically. No other tracks like it are present on the surface. An alternative interpretation is that the impression is the result of two or more overprinting theropod tracks, though if this is the case, these tracks are also not part of clear trackways. Unfortunately, the photogrammetric documentation was taken before the track was identified, and as such this track, being particularly small, is not present in the model in sufficient detail to be presented as a height map.

The track surface appears to have been subjected to some level of erosion/weathering, and track boundaries are not well defined in most cases for both the small and larger tracks. The relatively uniform direction of travel on the surface is reminiscent of the numerous small tracks from Lark Quarry, Australia [47,48], though here the larger trackway indicates movement in the same direction as the smaller tracks, rather than counter to it.

### 4.3. Tracksite three

#### 4.3.1. Sedimentology and palaeoenvironmental interpretation

The tracksite is located on the northern limb of the AAK syncline, not far from its southwestern perisynclinal termination. The footprints of this tracksite are well preserved on the surface of a horizontally laminated fine sandstone bed. The surface of the bed shows, in addition to footprints, small sedimentary structures resembling microbially induced sedimentary structures (*sensu* Noffke *et al*. [49]) and desiccation cracks, as well as burrows (figure 3). This centimetric bed is intercalated between grey silty argillites. These mudstones were deposited in the same palaeoenvironment as that described for tracksite two.

The sandstone beds indicate channel and sheetflood deposits, the fine sediments are floodplain and open lake deposits.

These sedimentological observations lead us to conclude that the depositional environment of these facies corresponds to a continental fluvio-lacustrine environment, like the palaeoenvironment of the other tracksites.

#### 4.3.2. Description of the track horizon

**Figure 7.**
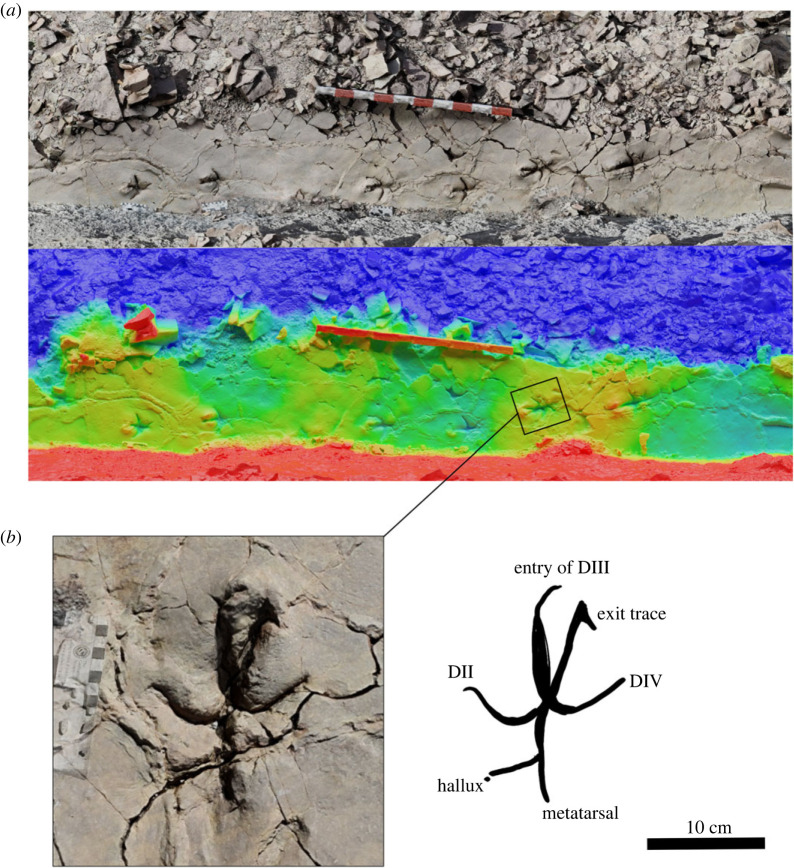
Tracks from tracksite 3, displaying penetrative nature and avian-like morphology. (*a*) True colour and height map of the complete trackway (red–blue scale = 10 cm). (*b*) Interpretation of one track, displaying both entry and exit traces, and raised area at the anterior of the track.

The track-bearing horizon is located about 3 km to the south of the Ait Ali Ou Ikkou village, at coordinates 32°7′19.06″ N; 5°35′24.48″ W. It is a centimetric bed of fine sandstone inclined at 40° to the south. The surface is a narrow bedding plane exposed for more than 5 m and is 30–60 cm wide. It contains a single 4 m long trackway, trending 210°, with nine deep and well-preserved bird-like footprints (figure 7). The tracks are tridactyl with extremely narrow digit impressions and a high interdigital angle, highly reminiscent of modern bird tracks. Each track is approximately 17 cm long, with a short stride length of approximately 42 cm. Some tracks preserve a posterior hallux impression, and all have a posterior elongation caused by indentation of the metatarsals [50].

This is the first site discovered in the Isli Formation that contains bird-like tracks. Gierliński *et al*. [2] described bird-like footprints in the Imilchil Formation (Upper Bajocian–Lower Bathonian) and referred to them as *Trisauropodiscus* isp. About 50 km southeast from this tracksite, at the locality of M'smrir, Belvedere *et al*. [51] documented similar tracks, of the same age (Lower Bathonian–?Upper Jurassic), and described them as *Anomoepus*-like.

The tracks visible at tracksite three are clearly penetrative in nature [52–55], where the foot has penetrated the original tracking surface, before being withdrawn. Several tracks show distinct entry and exit traces from digit III (figure 7), and probably record three-dimensional motions of the foot within the subsurface track volume [54,56]. While anatomical fidelity (foot pads, skin impressions) is lacking, the tracks are clearly exceptionally well preserved [52], appearing very similar to modern bird tracks made in soft mud. The short stride length is probably a result of the animal moving slowly over a deep, deformable substrate.

## 5. Discussion and conclusion

In a region such as Imilchil, where the palaeoenvironment was favourable for the preservation of the tracks and trackways of various tetrapod ichnofaunas forming an ichnoassemblage dominated by dinosaurs, the discovery of these three new tracksites increases our knowledge of the richness and diversity of the dinosaurs from Morocco in particular and North Africa in general.

Dinosaur body fossils are unknown from the Middle to Late Jurassic of the Imilchil area, and are rare across the whole of North Africa. To date, only four taxa have been described from the Middle to Late Jurassic of Morocco, all of which are either sauropodomorphs or thyreophorans.

‘*Cetiosaurus’ mogrebiensis* is from the El Mers 1 Formation (Bathonian) of the Middle Atlas [57], while *Atlasaurus imelakei* is from the Guettioua Formation (Bathonian–?Callovian) in the northern front of the Central High Atlas [58]; both are sauropodomorphs. The stegosaur *Adratiklit boulahfa* and the ankylosaur *Spicomellus afer* are both from the El Mers 3 Formation (Bathonian–Callovian) in the Middle Atlas [59,60]. In other North African countries, several additional Middle Jurassic dinosaur body fossils have been discovered, although all are sauropods: *Chebsaurus algeriensis* from the Aissa Formation (Callovian) in northwest Algeria [61,62], ‘*Brachiosaurus’ nougaredi* from the Middle Jurassic of Taouratine in eastern Algeria [63], *Spinophorosaurus* from the Irhazer Group (Bathonian–Callovian) in the Iullemmeden Basin of Niger [64] and *Jobaria* from Tiouaren Formation (Callovian) in the Iullemmeden Basin of Niger [65,66].

The tracks described here and previously from the Central High Atlas indicate that Middle to Late Jurassic ecosystems in Morocco were far more diverse than is indicated by the body fossil record. The dinosaurs that left these footprints were large sauropods, small and medium-sized theropods, and medium-sized ornithopods. To date, there is no body fossil record of ornithopods or theropods from the Middle to Late Jurassic of Morocco, although their tracks have been documented previously in the area [8]. The oldest bird body fossils worldwide are those of *Archaeopteryx* von Meyer [67] from the Upper Jurassic Solnhofen Limestone (Germany). In Morocco, bird-like tracks have previously been described in the uppermost levels of the Imilchil Formation and correlative deposits [2,51], though whether these were made by avian or non-avian theropods is unknown.

Body fossils cannot reveal the whole picture of fauna diversity, especially of dinosaurs in the Jurassic of Morocco, so ichnology is a powerful tool for reconstructing ecosystems. The discovery of these three tracksites complements the others already discovered in the region. As well as revealing the diversity of the dinosaurs, they also reveal information about the interaction between the behaviour of the dinosaurs and the substrate.

## Data Availability

All data (photographs and models) are available from https://doi.org/10.6084/m9.figshare.23374418 [68]. The data are provided in electronic supplementary material [69].

## References

[1] Gierliński GD, Menducki P, Janiszewska K, Wicik I, Boczarowski A. 2009 A preliminary report on dinosaur track assemblages from the Middle Jurassic of the Imilchil area, Morocco. Geological Q. **53**, 477-482.

[2] Gierliński GD, Lagnaoui A, Klein K, Saber H, Oukassou M, Charrière A. 2017 Bird-like tracks from the Imilchil Formation (Middle Jurassic, Bajocian-Bathonian) of the Central High Atlas, Morocco, in comparison with similar Mesozoic tridactylous ichnotaxa. Boll. Soc. Paleontol. Ital. **56**, 207-215. (10.4435/BSPI.2017.19)

[3] Klein H, Lagnaoui A, Gierliński GD, Saber H, Lallensack JN, Oukassou M, Charrière A. 2018 Crocodylomorph, turtle and mammal tracks in dinosaur-dominated Middle to Upper Jurassic and mid-Cretaceous ichnoassemblages of Morocco. Palaeogeogr. Palaeoclimatol. Palaeoecol. **498**, 39-52. (10.1016/j.palaeo.2018.02.028)

[4] Masrour M, Boutakiout M, Pérez-Lorente F. 2020 Footprints of *Batrachopus* isp. from the Imilchil megatracksite. Middle? - Upper Jurassic, central High Atlas (Morocco). J. Afr. Earth Sci. **172**, 103980. (10.1016/j.jafrearsci.2020.103980)

[5] Oukassou M et al. 2019 *Polyonyx*-like tracks from Middle-?Upper Jurassic red beds of Morocco: implications for sauropod communities on southern margins of tethys. Palaeogeogr. Palaeoclimatol. Palaeoecol. **536**, 109394. (10.1016/j.palaeo.2019.109394)

[6] Masrour M, Boutakiout M, Herrero Gascón J, Ochoa Martínez R, Sáinz Ruiz de Zuazu JL, Pérez-Lorente F. 2021 Crocodile tail traces and dinosaur footprints. Bathonian?-Callovian. Imilchil, High Central Atlas, Morocco. Geogacet **69**, 95-98.

[7] Ceniceros JM, Farlow JO, Masrour M, Extremiana JI, Boutakiout M, Pérez-Lorente F. 2022 Demographic interpretation of colossal theropod footprints discoveries from Imilchil (Mid-Jurassic, Central High Atlas, Morocco). J. Afr. Earth Sci. **193**, 104595. (10.1016/j.jafrearsci.2022.104595)

[8] Klein H, Gierliński GD, Oukassou M, Saber H, Lallensack JN, Lagnaoui A, Hminna A, Charrière A. 2023 Theropod and ornithischian dinosaur track assemblages from Middle to Late Jurassic deposits of the Central High Atlas, Morocco. Hist. Biol. **35**, 320-346. (10.1080/08912963.2022.2042808)

[9] Plateau H, Giboulet G, Roch E. 1937 Sur la présence d'empreintes de dinosauriens dans la région de Demnat (Maroc). C. R. Somm. Soc. Géol. Fr. **16**, 241-242.

[10] Dutuit JM, Ouazzou A. 1980 Découverte d'une piste de Dinosaure sauropode sur le site d'empreintes de Demnat (Haut-Atlas marocain). Mémoir. Soc. Géologique Fr. Nouv. série **59**, 95-102.

[11] Ishigaki S. 1985 Dinosaur footprints of the Atlas mountains. Nature Study. Osaka City Mus. Nat. Hist. **31**, 3-8.

[12] Ishigaki S. 1988 Les empreintes de dinosaures du Jurassique inférieur du Haut Atlas central marocain. Notes Serv. Géol. Maroc. **44**, 79-86.

[13] Ishigaki S. 1989 Footprints of swimming sauropods from Morocco. In Dinosaur tracks and traces (eds DD Gillette, MG Lockley), pp. 83-86. Cambridge, UK: Cambridge University Press.

[14] Ishigaki S, Matsumoto Y. 2009 'Off-tracking'-like phenomenon observed in the turning sauropod trackway from the Upper Jurassic of Morocco. Memoir Fukui Prefectural Dinosaur Mus. **8**, 1-10.

[15] Ishigaki S, Lockley MG. 2010 Didactyl, tridactyl and tetradactyl theropod trackways from the Lower Jurassic of Morocco: evidence of limping, labouring and other irregular gaits. Hist. Biol. **22**, 100-108. (10.1080/08912961003789867)

[16] Belvedere M, Mietto P, Ishigaki S. 2010 A Late Jurassic diverse ichnocoenosis from the siliciclastic Iouaridene Formation (Central High Atlas, Morocco). Geol. Q. **54**, 367-380.

[17] Belvedere M, Mietto P. 2010 First evidence of stegosaurian *Deltappodus* footprints in North Africa (Iouaridène Formation, Upper Jurassic. Morocco). Palaeontology **53**, 233-240. (10.1111/j.1475-4983.2009.00928.x)

[18] Nouri J, Díaz-Martínez I, Pérez-Lorente F. 2011 Tetradactyl footprints of an unknown affinity theropod dinosaur from the Upper Jurassic of Morocco. PLoS ONE **6**, e26882. (10.1371/journal.pone.0026882) 22180775 PMC3236743

[19] Boutakiout M, Herrero J, Ochoa R, Pereda JC, Sáinz JL, Pérez-Lorente F. 2019 Giant theropod footprints in the Upper Jurassic of Morocco. Aït Mazigh site (central Atlas). Geogaceta **66**, 83-86.

[20] Lallensack JN, Ishigaki S, Lagnaoui A, Buchwitz M, Wings O. 2019 Forelimb orientation and locomotion of sauropod dinosaurs: insights from the ?Middle Jurassic Tafaytour tracksites (Argana basin, Morocco). J. Vertebr. Paleontol. **38**, e1512501. (10.1080/02724634.2018.1512501)

[21] Teixell A, Arboleya ML, Julivert M, Charroud M. 2003 Tectonic shortening and topography in the central High Atlas (Morocco). Tectonics. **22**, 1051. (10.1029/2002TC001460)

[22] Mattauer M, Tapponnier P, Proust F. 1977 Sur les mécanismes de formation des chaines intracontinentales; l'exemple des chaines atlasiques du Maroc. Bull. Soc. géol. Fr. **7**, 521-526. (10.2113/gssgfbull.S7-XIX.3.521)

[23] Frizon de Lamotte D et al.. 2008 The Atlas system. In Continental evolution: the geology of Morocco (eds A Michard, O Saddiqi, A Chalouan, D Frizon de Lamotte), pp. 133-202. Lecture Notes in Earth Sciences, vol. 116. Berlin, Germany: Springer. (10.1007/978-3-540-77076-3_4)

[24] Ibouh A, Chafiki D. 2017 La tectonique de l'Atlas: âge et modalités. Géologues **194**, 24-28.

[25] Fekkak A, Ouanaimi H, Michard A, Soulaimani A, Ettachfini EM, Berrada I, El Arabi H, Lagnaoui A, Saddiqi O. 2018 Thick-skinned tectonics in a Late Cretaceous Neogene intracontinental belt (High Atlas Mountains, Morocco): the flat-ramp fault control on basement shortening and cover folding. J. Afr. Earth Sci. **140**, 169-188. (10.1016/j.jafrearsci.2018.01.008)

[26] Marzoli A, Renne PR, Piccirillo EM, Ernesto M, Bellieni G, De Min A. 1999 Extensive 200 million-year-old continental flood basalts of the Central Atlantic Magmatic Province. Science **284**, 616-618. (10.1126/science.284.5414.616) 10213679

[27] Marzoli H et al. 2019 The Central Atlantic Magmatic Province (CAMP) in Morocco. J. Petrol. **60**, 945-996. (10.1093/petrology/egz021)

[28] Ibouh H, Saidi A, Bouabdelli M, Youbi N, Boummane K, Aït Chayeb EH. 2002 Les roches volcaniques triasico-liasiques du Maroc; exemple de la ride de Tasraft (Haut Atlas central), données pétrologiques, géochimiques et implications géodynamiques. Africa Geosci. Rev. **9**, 75-92.

[29] Panfili G, Cirilli S, Dal Corso J, Bertrand H, Medina F, Youbi N, Marzoli A. 2019 New palynological constraints show rapid emplacement of the Central Atlantic Magmatic Province (CAMP) during the end-Triassic mass extinction interval. Glob. Planet. Change **172**, 60-68. (10.1016/j.gloplacha.2018.09.009)

[30] Armando G. 1999 Intracontinental alkaline magmatism: geology, petrography, mineralogy and geochemistry of the Jebel Hayim Massif (central High Atlas-Morocco). Mém. Géol. Lausanne. **31**, 1–106.

[31] Zayane R, Essaifi A, Maury RC, Piqué A, Laville E, Bouabdelli M. 2002 Cristallisation fractionnée et contamination crustale dans la série magmatique jurassique transitionnelle du Haut Atlas central (Maroc). C. R. Geosci. **334**, 97-104. (10.1016/S1631-0713(02)01716-9)

[32] Barbero L, Teixell A, Arboleya ML, Del Río P, Reiners PW, Bougadir B. 2007 Jurassic-to-present thermal history of the central High Atlas (Morocco) assessed by low-temperature thermochronology. Terra Nova **19**, 58-64. (10.1111/j.1365-3121.2006.00715.x)

[33] Calvín P, Ruiz-Martínez VC, Villalaín JJ, Casas-Sainz AM, Moussaid B. 2017 Emplacement and deformation of Mesozoic Gabbros of the High Atlas (Morocco): paleomagnetism and magnetic fabrics. Tectonics **36**, 3012-3037. (10.1002/2017TC004578)

[34] Essaifi A, Zayane R. 2018 Petrogenesis and origin of the Upper Jurassic-Lower Cretaceous magmatism in Central High Atlas (Morocco): major, trace element and isotopic (Sr-Nd) constraints. J. Afr. Earth Sci. **137**, 229-245. (10.1016/j.jafrearsci.2017.10.002)

[35] Charrière A, Haddoumi H, Mojon P-O, Ferrière J, Cuche D, Zili L. 2009 Mise en évidence par charophytes et ostracodes de l’âge Paléocène des dépôts discordants sur les rides anticlinales de la région d'Imilchil (Haut Atlas, Maroc): conséquences paléogéographiques et structurales. C. R. Palevol. **8**, 9-19. (10.1016/j.crpv.2008.11.006)

[36] Michard A, Ibouh H, Charrière A. 2011 Syncline-topped anticlinal ridges from the High Atlas: a Moroccan conundrum, and inspiring structures from the Syrian Arc, Israel. Terra Nova **23**, 314-323. (10.1111/j.1365-3121.2011.01016.x)

[37] Studer MR. 1987 Tectonique et Pétrographie des roches sédimentaires, éruptives et métamorphiques de la région de Tounfite-Tirrhist. (Haut Atlas central, Mésozoïque, Maroc). Not. Mem. Serv. Géol. Maroc. **43**, 65-197.

[38] Charrière A, Ibouh H, Haddoumi H. 2011 Circuit C7, Le Haut Atlas central de Beni Mellal à Imilchil. In *Nouveaux guides géologiques et miniers du Maroc* (eds A Michard, O Saddiqi, A Chalouan, A Mouttaqi). Not. Mém. Serv. Géol. Maroc. **559**, 109-164.

[39] Ibouh H, Michard A, Charrière A, Benkaddour A, Rhoujjati A. 2014 Tectono-karstic origin of the alleged ‘impact crater’ of Lake Isli (Imilchil district, High Atlas, Morocco). C. R. Geos. **346**, 82-89. (10.1016/j.crte.2014.03.005)

[40] Ibouh H. 2004 Du rift avorté au bassin sur décrochement, contrôles tectonique et sédimentaire pendant le Jurassique (Haut Atlas central, Maroc). PhD thesis, Cadi Ayyad University, Marrakech, Morocco.

[41] Falkingham PL et al. 2018 A standard protocol for documenting modern and fossil ichnological data. Palaeontology **61**, 469-480. (10.1111/pala.12373)

[42] Gatesy SM, Falkingham PL. 2017 Neither bones nor feet: track morphological variation and ‘preservation quality’. J. Vertebr. Paleontol. **37**, e1314298. (10.1080/02724634.2017.1314298)

[43] Falkingham PL, Gatesy SM. 2020 Discussion: defining the morphological quality of fossil footprints. Problems and principles of preservation in tetrapod ichnology with examples from the Palaeozoic to the present by Lorenzo Marchetti *et al*. Earth-Sci. Rev. **208**, 103320. (10.1016/j.earscirev.2020.103320)

[44] Belvedere M, Farlow JO. 2016 A numerical scale for quantifying the quality of preservation of vertebrate tracks. In Dinosaur tracks: the next steps (eds PL Falkingham, D Marty, A Richter), pp. 93-99. Bloomington, IL: Indiana University Press.

[45] Manning PL. 2004 A new approach to the analysis and interpretation of tracks: examples from the Dinosauria. In The application of ichnology to palaeoenvironmental and stratigraphic analysis (ed. D McIlro), pp. 93-123. Special Publications vol. 228. London, UK: Geological Society.

[46] Boutakiout M, Masrour M, Pérez-Lorente F. 2020 New sauropod morphotype definition in the oriental section of Imilchil megatracksite, High Atlas (Morocco). J. Afr. Earth Sci. **161**, 103664. (10.1016/j.jafrearsci.2019.103664)

[47] Thulborn T, Wade M. 1984 Dinosaur trackways in the Winton Formation (Mid-Cretaceous) of Queensland. Mem. Queensl. Mus. **21**, 413-517.

[48] Thulborn RA, Wade M. 1979 Dinosaur stampede in the Cretaceous of Queensland. Lethaia **12**, 275-279. (10.1111/j.1502-3931.1979.tb01008.x)

[49] Noffke N, Gerdes G, Klenke T, Krumbein WE. 2001 Microbially induced sedimentary structures: a new category within the classification of primary sedimentary structures. J. Sediment. Res. **71**, 649-656. (10.1306/2DC4095D-0E47-11D7-8643000102C1865D)

[50] Lallensack JN, Farlow JO, Falkingham PL. 2021 A new solution to an old riddle: elongate dinosaur tracks explained as deep penetration of the foot, not plantigrade locomotion. Palaeontology **65**, e12584. (10.1111/pala.12584)

[51] Belvedere M, Dyke G, Hadri M, Ishigaki S. 2011 The oldest evidence for birds in northern Gondwana? Small tridactyl footprints from the Middle Jurassic of Msemrir (Morocco). Gondwana Res. **19**, 542-549. (10.1016/j.gr.2010.08.004)

[52] Gatesy SM, Falkingham PL. 2020 Hitchcock's Leptodactyli, penetrative tracks, and dinosaur footprint diversity. J. Vertebr. Paleontol. **40**, e1781142. (10.1080/02724634.2020.1781142)

[53] Turner ML, Falkingham PL, Gatesy SM. 2022 What is stance phase on deformable substrates? Integr. Comp. Biol. **62**, 1357-1368. (10.1093/icb/icac009) 35325150

[54] Falkingham PL, Turner ML, Gatesy SM. 2020 Constructing and testing hypotheses of dinosaur foot motions from fossil tracks, using digitization and simulation. Palaeontology **63**, 1-16. (10.1111/pala.12502)

[55] Lockley MG, Matsukawa M, Witt D. 2006 Giant theropod tracks from the Cretaceous Dakota group of northeastern New Mexico. New Mexico Mus. Nat. Hist. Sci. Bull. **35**, 83-87.

[56] Gatesy SM, Middleton KM, Jenkins FA, Shubin NH. 1999 Three-dimensional preservation of foot movements in Triassic theropod dinosaurs. Nature **399**, 141-144. (10.1038/20167)

[57] de Lapparent AF. 1955 Etude paléontologique des vertébrés du Jurassique d'El Mers (Moyen Atlas). Not. Mém. Serv. Géol. Maroc. **124**, 1-36.

[58] Monbaron M, Russell DA, Taquet P. 1999 *Atlasaurus imelakei*, n.g., n.sp., a brachiosaurid-like sauropod from the Middle Jurassic of Morocco. C. R. Acad. Sci. **329**, 519-526. (10.1016/S1251-8050(00)80026-9)

[59] Maidment SC, Raven TJ, Ouarhache D, Barrett PM. 2020 North Africa's first stegosaur: implications for Gondwanan thyreophoran dinosaur diversity. Gondwana Res. **77**, 82-97. (10.1016/j.gr.2019.07.007)

[60] Maidment SC, Strachan SJ, Ouarhache D, Scheyer TM, Brown EE, Fernandez V, Johanson Z, Raven TJ, Barrett PM. 2021 Bizarre dermal armour suggests the first African ankylosaur. Nat. Ecol. Evol. **5**, 1576-1581. (10.1038/s41559-021-01553-6) 34556830

[61] Mahammed F et al. 2005 The ‘Giant of Ksour,’ a Middle Jurassic sauropod dinosaur from Algeria. C. R. Palevol. **4**, 707-714. (10.1016/j.crpv.2005.07.001)

[62] Läng E, Mahammed F. 2010 New anatomical data and phylogenetic relationships of *Chebsaurus algeriensis* (Dinosauria, Sauropoda) from the Middle Jurassic of Algeria. Hist. Biol. **22**, 142-164. (10.1080/08912960903515570)

[63] de Lapparent AF. 1960 Les Dinosauriens du « Continental Intercalaire du Sahara central ». Mém. Soc. Géol. Fr. Nouv. série, Mém. n888A **39**, 56 p, XI pl.

[64] Remes K, Ortega F, Fierro I, Joger U, Kosma R, Ferrer JMM, Paldes Snhm Ide OA, Maga A. 2009 A new basal sauropod dinosaur from the Middle Jurassic of Niger and the early evolution of Sauropoda. PLoS ONE **4**, e6924. (10.1371/journal.pone.0006924) 19756139 PMC2737122

[65] Sereno PC et al. 1999 Cretaceous sauropods from the Sahara and the uneven rate of skeletal evolution among dinosaurs. Science **286**, 1342-1347. (10.1126/science.286.5443.1342) 10558986

[66] Rauhut OWM, López-Arbarello A. 2009 Considerations on the age of the Tiouaren Formation (Iullemmeden Basin, Niger, Africa): implications for Gondwanan Mesozoicterrestrial vertebrate faunas. Palaeogeogr. Palaeoclimatol. Palaeoecol. **271**, 259-267. (10.1016/j.palaeo.2008.10.019)

[67] von Meyer H. 1861 *Archaeopteryx lithographica* (Vogel-Feder) und *Pterodactylus* von Solenhofen. N. Jb. Geol. Paläont. Mh. Jahr. **1861**, 678-679.

[68] Oussou A, Falkingham PL, Butler RJ, Boumir K, Ouarhache D, Ech-charay K, Charrière A, Maidment SCR. 2023 Data from: New Middle to ?Late Jurassic dinosaur tracksites in the Central High Atlas Mountains, Morocco. Figshare. (10.6084/m9.figshare.23374418) PMC10523064 37771967

[69] Oussou A, Falkingham PL, Butler RJ, Boumir K, Ouarhache D, Ech-charay K, Charrière A, Maidment SCR. 2023 New Middle to ?Late Jurassic dinosaur tracksites in the Central High Atlas Mountains, Morocco. Figshare. (10.6084/m9.figshare.c.6845602) PMC10523064 37771967

